# Insulin Receptor Substrate p53 Ameliorates High-Glucose-Induced Activation of NF-*κ*B and Impaired Mobility of HUVECs

**DOI:** 10.1155/2021/3210586

**Published:** 2021-01-06

**Authors:** Fen Liu, Yubin Chen, Shi Zhao, Mei Li, Fanyan Luo, Can-e Tang

**Affiliations:** ^1^The Institute of Medical Science Research, Xiangya Hospital, Central South University, Changsha, 410008 Hunan, China; ^2^Department of Cardiac Surgery, Xiangya Hospital, Central South University, Changsha, 410008 Hunan, China

## Abstract

Diabetes-related macrovascular and microvascular complications lead to poor prognosis. Insulin receptor substrate p53 (IRSp53) is known to act as a substrate for the insulin receptor tyrosine kinase, but its role in endothelial dysfunction remains unclear. Human umbilical vein endothelial cells (HUVECs) treated with D-glucose at different concentrations and a streptozocin-induced rat diabetes mellitus (DM) model were used to investigate the effects of hyperglycemia on the expression levels of IRSp53 and galectin-3 (gal-3) and the inflammatory state and mobility of HUVECs. Thereafter, IRSp53-overexpressing HUVECs and IRSp53-knockdown HUVECs were established using IRSp53-overexpressing lentivirus or IRSp53-siRNA to explore the role of IRSp53 in the HUVEC inflammatory state and HUVEC mobility. D-glucose at high concentration (HG) and hyperglycemia were found to induce downregulation of IRSp53 and upregulation of gal-3 *in vitro* and *in vivo*. Treatment with HG resulted in activation of NF-*κ*B in HUVECs and impaired HUVEC mobility. Insulin restored HG-induced changes in the expression levels of IRSp53 and gal-3 in HUVECs and protected the cells from NF-*κ*B activation and impaired mobility. Overexpression of IRSp53 inhibited the activation of NF-*κ*B in HUVECs and strengthened HUVEC migration. Knockdown of IRSp53 facilitated the activation of NF-*κ*B in HUVECs and decreased HUVEC migration. However, neither overexpression nor knockdown of IRSp53 altered the effects of insulin on HG-induced detrimental changes in HUVECs. HG and hyperglycemia resulted in downregulation of IRSp53 *in vitro* and *in vivo*. IRSp53 is concluded to inhibit the activation of NF-*κ*B in HUVECs and to strengthen HUVEC migration.

## 1. Introduction

The number of patients with diabetes mellitus (DM) is rapidly increasing due to modern lifestyle, gene susceptibility, and aging [1]. Researchers have predicted a 4.4% prevalence of DM worldwide by 2030 [2]. Diabetes-related macrovascular complications such as coronary atherosclerosis lead to severe cardiac ischemia [3], and microvascular complications such as retinopathy and nephropathy can result in blindness and renal failure [4]. Endothelial dysfunction characterized by vasodilation dysfunction, impaired endothelial cell migration capability, and proinflammatory changes in endothelial cells composes the early stage of diabetes-related macrovascular and microvascular complications [5]. Hyperglycemia contributes to endothelial dysfunction [6], but the exact mechanism underlying hyperglycemia-induced endothelial dysfunction remains unknown.

Insulin receptor substrate p53 (IRSp53) is a crucial regulator of membrane curvature, filopodium formation, and the actin cytoskeleton in mammalian cells [7]. IRSp53 induces the formation of dendritic spines in neurons [8], and downregulation of IRSp53 is associated with severe learning deficits [9], autism [10], and schizophrenia [11]. Furthermore, IRSp53 is important for the metastatic behavior of malignant tumor cells [12] and can provoke growth and mobility of cancer cells [13]. In addition to the formation of dendritic spines in neurons and mobility enhancement of cancer cells, Kaur et al. reported that IRSp53 interacted with cell division cycle 42 (Cdc42), a guanosine triphosphate binding protein, to induce the formation of filopodia and migration of endothelial cells [14] which is essential for homeostasis of the cardiovascular system [15]. Whether IRSp53 plays a role in hyperglycemia-induced endothelial dysfunction, however, needs further exploration.

Galectin-3 (gal-3), a member of the galectin family, contains two domains, namely, the C-terminal carbohydrate recognition domain and the N-terminal domain, and is associated with a variety of diseases including cancer, inflammatory diseases, and DM [16]. In terms of cellular function, gal-3 is involved in cell growth, differentiation, apoptosis, and migration [17]. Yilmaz et al. reported that the expression level of gal-3 in patients with DM was significantly higher than that in patients with prediabetes or in patients without DM [18]. Gal-3 was found to cause cellular and systemic insulin resistance [19] and was independently associated with nephropathy in patients with DM—the predominant microvascular complication of DM [20]. However, it is not clear whether a relationship exists between IRSp53 and gal-3 in hyperglycemia-induced endothelial dysfunction.

In the present study, human umbilical vein endothelial cells (HUVECs) treated with different concentrations of glucose and a streptozocin-induced rat DM model were used to investigate the role of IRSp53 in hyperglycemia-induced endothelial dysfunction, the relationship between IRSp53 and gal-3, and the effect of insulin on hyperglycemia-induced endothelial dysfunction.

## 2. Materials and Methods

### 2.1. Cell Culture, Treatment, and Transfection

HUVECs were kindly provided by Professor Bai and cultured in Dulbecco's modified Eagle's medium (DMEM, HyClone) containing 10% fetal bovine serum (FBS) and 1% penicillin-streptomycin. HUVECs from passages 3 to 8 were used in this study. The appropriate glucose concentration and treatment time were determined by treating HUVECs with D-glucose (G7021, Sigma-Aldrich) at the following concentrations: 25 mM D-glucose (normal glucose group (NG)), 40 mM D-glucose (H1 group), 60 mM D-glucose (H2 group), and 80 mM D-glucose (H3 group) for 24, 48, 72, and 96 h. In addition, HUVECs were treated with 25 mM D-glucose+60 mM mannitol to exclude the effect of the osmotic pressure of 80 mM D-glucose. In the following experiments, HUVECs were treated for 96 h with D-glucose and/or insulin (I0908, Sigma-Aldrich) at the following concentrations: 25 mM D-glucose (normal glucose group (NG)), 60 mM D-glucose (high-glucose group (HG)), and 60 mM D-glucose+1.5 *μ*g/mL insulin (HG+INS group). HUVECs were transfected with IRSp53-siRNA or IRSp53-overexpressing lentivirus according to the manufacturers' recommended procedures. Descriptions of the IRSp53-siRNA- and IRSp53-overexpressing lentiviruses appear in the Supplementary Information (available here).

### 2.2. Establishment of a Streptozocin-Induced Rat DM Model

All experimental procedures were conducted in accordance with the guidelines of the Animal Welfare Committee of Central South University. Sixteen male SD rats were purchased from the Experimental Animal Department of Central South University. The rats were randomly divided into two groups: a normal control (NC) group (*n* = 6) and a DM group (*n* = 10). To establish a streptozocin-induced rat DM model, rats in the DM group were treated with a single intraperitoneal injection of streptozocin (STZ, S0130, Sigma-Aldrich) at a concentration of 60 mg/kg in citrate buffer (pH 4.5) after 12 h of food deprivation. Blood glucose levels were determined at 3 days after injection of STZ using a glucometer (Accu-Chek, Roche). Rats with constant high blood glucose concentrations of ≥16.7 mmol/L for 3 consecutive days were used in the study. Rats in the normal control group were treated with a single intraperitoneal injection of citrate buffer after 12 h of food deprivation.

### 2.3. Western Blotting

Rat aortas were homogenized in radioimmunoprecipitation assay (RIPA) buffer (P0013B, Beyotime) containing 1% phenylmethylsulphonyl fluoride (PMSF, ST506, Beyotime) using TissueLyser (Qiagen), and HUVECs were lysed in RIPA buffer containing 1% PMSF. The suspension of the aortas or HUVECs were centrifuged at 12000 g at 4°C for 30 min, and the supernatant was collected. A bicinchoninic acid (BCA) assay (23227, Thermo Fisher Scientific) was used to determine the protein concentration of the supernatant. Then, 20 *μ*g protein was separated on 10% Bis-Tris gels and transferred to polyvinylidene fluoride (PVDF) membranes (IPVH00010, Millipore). The membranes were blocked in 5% bovine serum albumin at 25°C for 120 min and incubated overnight at 4°C with primary antibodies against the following proteins: *β*-actin (1 : 5000 dilution, AF7018, Affinity Biosciences), IRSp53 (1 : 1000 dilution, ab126057, Abcam), gal-3 (1 : 1500 dilution, ab2785, Abcam), nuclear factor kappa B (NF-*κ*B, 1 : 1000 dilution, #6956, CST), and NF-*κ*B inhibitor *α* (I*κ*B*α*) (1 : 1000 dilution; #4812, CST). Subsequently, the PVDF membranes were incubated with corresponding horseradish peroxidase- (HRP-) conjugated secondary antibodies (1 : 8000 dilution, #S0001, #S0002, Affinity Biosciences) at 25°C for 90 min and observed using an Enhanced Chemiluminescent Kit (p10100, NCMBiotech) on a ChemiDoc XRS+ (Bio-Rad) tool. Relative expression levels of protein were analyzed using Image Lab 3.0 software.

### 2.4. Immunofluorescence

Formalin-fixed, paraffin-embedded aorta sections (4 *μ*m) were deparaffinized, hydrated, and antigen retrieved. Sections were then incubated with primary antibodies against CD31 (1 : 150 dilution, GB12063, Servicebio) and IRSp53 (1 : 150 dilution, DF3853, Affinity Biosciences) or gal-3 (1 : 150 dilution, PB9081, Boster Biological Technology) overnight at 4°C. After washing thrice with PBS, sections were incubated with Alexa Fluor 488 goat-anti-rabbit IgG H&L (1 : 600 dilution, ab150077, Abcam) and Alexa Fluor 594 donkey-anti-mouse IgG H&L (1 : 600 dilution, 715-585-150, Jackson Laboratories) at 25°C for 40 min. Then, sections were stained with 4′,6-diamidino-2-phenylindole (DAPI, 1 : 1000 dilution, 564907, BD Pharmingen Inc.) at 25°C for 5 min and washed thrice with PBS. The images (20x magnification) were obtained using a Vectra Polaris integrated automatic multispectral scanning imaging system (Akoya Biosciences), and the images were analyzed using inForm software (Akoya Biosciences). The area which expressed CD31 and IRSp53 or CD31 and gal-3 simultaneously in the intima of aorta was measured and the colocalization ratios were calculated: CD31 − and IRSp53 − positive area in intima of aorta/CD31 area in intima of aorta; CD31 − and gal − 3 − positive area in intima of aorta/CD31 area in intima of aorta.

After treatment, the HUVECs were fixed using 4% paraformaldehyde for 25 min, permeated using 0.5% Triton X-100 for 20 min, and incubated with primary antibodies against NF-*κ*B (1 : 200 dilution, #6956, CST) overnight at 4°C. After washing thrice with PBS, the HUVECs were incubated with Alexa Fluor 594 donkey-anti-mouse IgG H&L (1 : 600 dilution, 715-585-150, Jackson Laboratories) at 25°C for 40 min. The HUVECs were washed thrice with PBS again, stained with DAPI (1 : 1000 dilution; 564907, BD Pharmingen) at 25°C for 5 min, and then washed thrice with PBS once again. Cellular immunofluorescence images were obtained by microscopy (DM5000 B, Leica).

### 2.5. Scratch Assay

HUVECs (after different treatments) were seeded in 6-well plates at 5 × 10^5^ cells/well. Following cell adherence, the surface of each well was carefully scratched using 10 *μ*L pipette tips. The HUVECs were washed thrice with PBS to remove cell debris and cultured in nonserum DMEM. Scratch assay images were obtained using microscopy (DM5000 B, Leica) at 0 h and 48 h after scratching. Image-Pro Plus 6.0 software was used to analyze the blank area of each image, and the migration rate was calculated as follows: (initial blank area − blank area at 48 h)/initial blank area.

### 2.6. Migration Assay

A 24-well modified Boyden chamber (8 *μ*m, Corning) was used to investigate HUVEC mobility. After different treatments, HUVECs were collected and resuspended in nonserum DMEM. Approximately 5 × 10^4^ cells in 200 *μ*L nonserum DMEM were seeded in the upper chamber, and 600 *μ*L DMEM containing 10% FBS was added to the lower chamber. The HUVECs were then incubated in a 37°C incubator for 48 h. Subsequently, cells on the upper chamber were wiped away, and cells that had migrated to the lower face were fixed using 4% paraformaldehyde for 25 min and stained with crystal violet solution (C0121, Beyotime) for 5 min. Migration assay images were acquired using microscopy (Leica DM5000 B). The number of migrated cells was counted in three random fields using Image-Pro Plus 6.0 software.

### 2.7. Statistical Analysis

Data are presented as the mean ± standard deviation (SD). Data were compared by an unpaired Student *t*-test or one-way analysis of variance (ANOVA) using SPSS 19.00 software. Values of *P* < 0.05 were considered statistically significant.

## 3. Results

### 3.1. A High Concentration of D-Glucose Affected the Expression Levels of IRSp53 and Gal-3 in HUVECs

**Figure 1:**
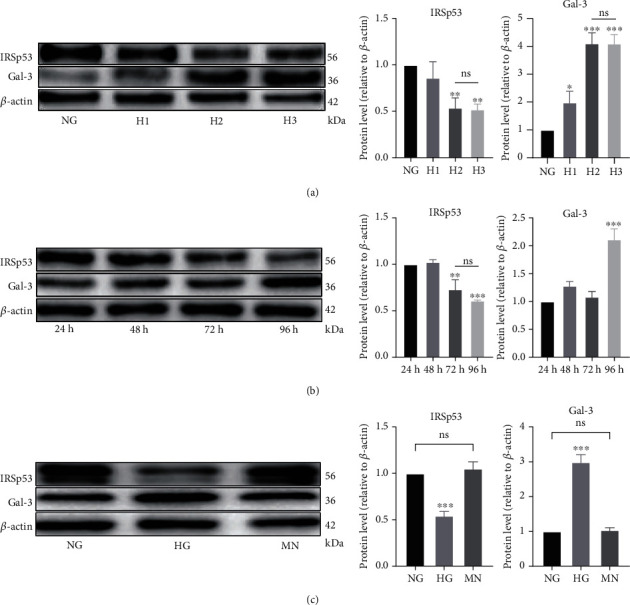
A high D-glucose concentration affected the expression levels of IRSp53 and gal-3 in HUVECs. (a) The expression levels of IRSp53 and gal-3 in HUVECs treated with D-glucose at different concentration for 96 h were determined by Western blotting using *β*-actin as a loading control. NG: normal glucose (25 mM); H1: high glucose 1 (40 mM); H2: high glucose 2 (60 mM); H3: high glucose 3 (80 mM). (b) The expression levels of IRSp53 and gal-3 in HUVECs treated with 60 mM D-glucose for different times were determined by Western blotting using *β*-actin as a loading control. (c) The expression levels of IRSp53 and gal-3 in HUVECs treated with 60 mM D-glucose or 60 mM mannitol for 96 h were determined by Western blotting using *β*-actin as a loading control. NG: normal glucose (25 mM); HG: high glucose (60 mM); MN: mannitol (60 mM). The values are the mean ± SD of three independent experiments. ^∗^*P* < 0.05 vs. the NG group; ^∗∗^*P* < 0.01 vs. the NG group; ^∗∗∗^*P* < 0.001 vs. the NG group.

To explore the effect of a high concentration of D-glucose (HG) on HUVECs, the cells were treated with D-glucose at concentrations of 25 mM (NG), 40 mM (H1), 60 mM (H2), and 80 mM (H3) for 96 h. The results of Western blotting demonstrated that HUVECs treated with 60 mM and 80 mM D-glucose exhibited significant downregulation of IRSp53 compared with HUVECs treated with 25 mM D-glucose and that the expression level of IRSp53 showed no significant difference between treatments of 60 mM and 80 mM D-glucose (Figure 1(a)). Moreover, the expression level of gal-3 was significantly upregulated in HUVECs treated with 60 mM and 80 mM D-glucose compared with that of HUVECs treated with 25 mM D-glucose, and the expression level of gal-3 showed no significant difference between 60 mM and 80 mM D-glucose treatment (Figure 1(a)). Thus, a glucose concentration of 60 mM was used in the subsequent experiments.

The results above indicate that HG affected the expression levels of IRSp53 and gal-3. The study continued to explore the effect of a high D-glucose exposure time on cells. HUVECs were treated with 60 mM D-glucose for 24, 48, 72, and 96 h. The results of Western blotting showed that the expression level of IRSp53 was significantly downregulated after treatment with 60 mM D-glucose for 72 and 96 h, while the expression level of gal-3 was significantly increased after treatment with 60 mM D-glucose for 96 h (Figure 1(b)).

High concentrations of D-glucose are accompanied by high osmotic pressures. To exclude the effect of high osmotic pressure on the HUVECs, the cells were treated with 60 mM mannitol, which has an equivalent osmotic pressure to 60 mM D-glucose. The results of Western blotting suggested that a high osmotic pressure did not significantly affect the expression levels of IRSp53 or gal-3 in the HUVECs (Figure 1(c)).

### 3.2. DM-Induced Changes in the Expression Levels of IRSp53 and Gal-3 in the Rat Aorta

**Figure 2:**
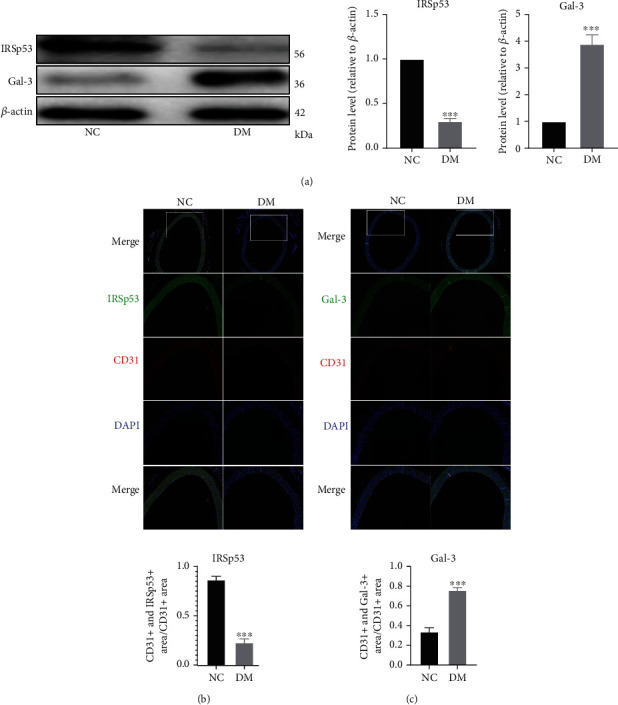
DM-induced changes in the expression levels of IRSp53 and gal-3 in the aorta of rats. (a) The expression levels of IRSp53 and gal-3 in the aorta of NC rats or DM rats were investigated by Western blotting using *β*-actin as a loading control (NC group, *n* = 6; DM group, *n* = 10). (b) Representative images of aorta immunofluorescence staining showing levels of IRSp53 and CD31 (20x magnification) (NC group, *n* = 6; DM group, *n* = 10); the colocalization ratio of IRSp53 is shown in the bar graph. (c) Representative images of aorta immunofluorescence staining showing levels of gal-3 and CD31 (20x magnification) (NC group, *n* = 6; DM group, *n* = 10); the colocalization ratio of IRSp53 is shown in the bar graph. ^∗∗∗^*P* < 0.001 vs. the NC group.

A STZ-induced DM rat model was established to further explore the effect of hyperglycemia on the expression levels of IRSp53 and gal-3. Western blotting demonstrated that the expression level of IRSp53 was significantly decreased in the aortas of DM rats compared with those of control rats, while the expression level of gal-3 was significantly higher (Figure 2(a)). Then, cellular immunofluorescence images of the aorta were obtained to determine the changes in the expression levels of IRSp53 and gal-3 in the endothelial layer of the aorta. The results demonstrated that the colocalization ratio of IRSp53 was significantly decreased in DM rats compared with that of NC rats (Figure 2(b)), while the colocalization ratio of gal-3 was significantly increased in DM rats compared with that of NC rats (Figure 2(c)). These results indicated that the expression level of IRSp53 in the endothelial layer was much lower in DM rats compared with that of NC rats and the expression level of gal-3 in the endothelial layer was significantly greater in DM rats than in NC rats.

### 3.3. Treatment with Insulin Restored HG-Induced Changes in the Expression Levels of IRSp53 and Gal-3 of HUVECs

**Figure 3:**
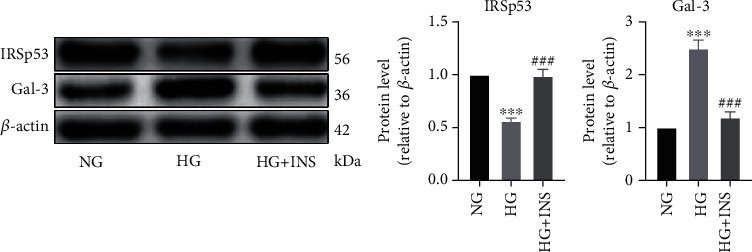
Treatment with insulin restored HG-induced changes in the HUVEC expression levels of IRSp53 and gal-3. (a) The expression levels of IRSp53 and gal-3 in HUVECs treated with D-glucose at different concentrations and/or 1.5 *μ*g/mL insulin were investigated by Western blotting using *β*-actin as a loading control. NG: normal glucose (25 mM); HG: high glucose (60 mM); HG+INS: high glucose (60 mM) + insulin (1.5 *μ*g/mL). The values are the mean ± SD of three independent experiments. ^∗∗∗^*P* < 0.001 vs. the NG group; ^###^*P* < 0.001 vs. the HG group.

HUVECs exposed to different concentrations of D-glucose were treated with or without 1.5 *μ*g/mL insulin. Changes in the expression levels of IRSp53 and gal-3 were determined by Western blotting, and the results suggested that insulin could upregulate the expression level of IRSp53, which was significantly decreased in the HUVECs exposed to HG while downregulating the HG-induced upregulation of gal-3 in HUVECs (Figure 3(a)).

### 3.4. The Role of IRSp53 in the Expression Level of Gal-3 and Inflammatory State of HUVECs

**Figure 4:**
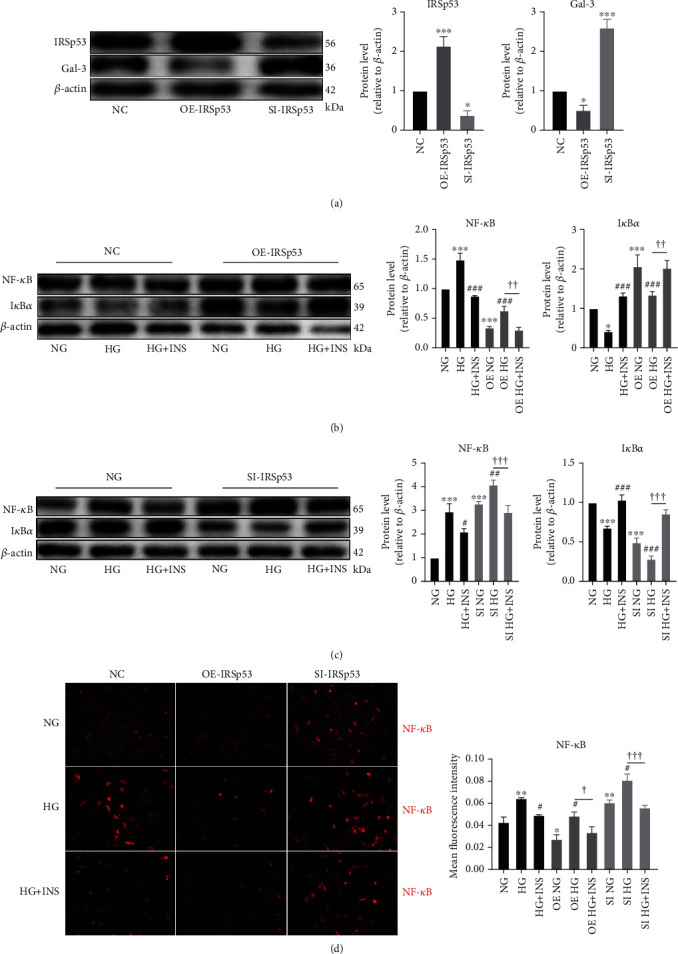
The role of IRSp53 in the expression level of gal-3 and inflammatory state of HUVECs. (a) The expression levels of IRSp53 and gal-3 in normal control (NC) HUVECs, HUVECs transfected with IRSp53 overexpressing lentivirus, and HUVECs transfected with IRSp53-siRNA were detected by Western blotting using *β*-actin as the loading control. (b) The expression levels of NF-*κ*B and I*κ*B*α* in NC HUVECs and IRSp53-overexpressing HUVECs with different treatments were determined by Western blotting using *β*-actin as the loading control. (c) The expression levels of NF-*κ*B and I*κ*B*α* in NC HUVECs and IRSp53-knockdown HUVECs with different treatments were investigated by Western blotting using *β*-actin as the loading control. (d) Representative images of cellular immunofluorescence staining of NF-*κ*B in HUVECs with different treatments (200x magnification). NC: normal control; OE-IRSp53: overexpressing IRSp53; SI-IRSp53: knockdown IRSp53; normal glucose (25 mM); HG: high glucose (60 mM); HG+INS: high glucose (60 mM) + insulin (1.5 *μ*g/mL). The values are the mean ± SD of three independent experiments. ^∗^*P* < 0.05 vs. the NG group; ^∗∗∗^*P* < 0.001 vs. the NG group; ^###^*P* < 0.001 vs. the HG group; ^††^*P* < 0.01; ^†††^*P* < 0.001.

To characterize the effects of IRSp53 on HUVECs, IRSp53-overexpressing HUVECs and IRSp53-knockdown HUVECs were established using IRSp53-overexpressing lentivirus or IRSp53-siRNA. HUVECs transfected with IRSp53-overexpressing lentivirus exhibited significant upregulation of IRSp53, while HUVECs transfected with IRSp53-siRNA showed significant IRSp53 downregulation (Figure 4(a)). The expression level of gal-3 was significantly increased in IRSp53-knockdown HUVECs but significantly decreased in IRSp53-overexpressing HUVECs (Figure 4(a)).

After HG treatment, HUVECs exhibited upregulation of NF-*κ*B and downregulation of I*κ*B*α*, indicating NF-*κ*B activation and a proinflammatory state. Cotreatment with insulin ameliorated HG-induced upregulation of NF-*κ*B and downregulation of I*κ*B*α*, which relieved the proinflammatory state (Figure 4(b)). Thus, the role of IRSp53 in the HG-induced inflammatory state of HUVECs was explored, and the results demonstrated that the expression level of NF-*κ*B was significantly decreased, while that of I*κ*B*α* was significantly increased in IRSp53-overexpressing HUVECs compared with control HUVECs. When IRSp53-overexpressing HUVECs were treated with HG, the expression level of NF-*κ*B was significantly downregulated and that of I*κ*B*α* was significantly upregulated compared with the case of control HUVECs treated with HG (Figure 4(b)). This indicates that overexpression of IRSp53 can effectively ameliorate the HG-induced inflammatory state of HUVECs. In contrast, significant upregulation of NF-*κ*B and downregulation of I*κ*B*α* were found in IRSp53-knockdown HUVECs compared with the case of control HUVECs (Figure 4(c)). Following treatment with HG, IRSp53-knockdown HUVECs exhibited significantly increased expression levels of NF-*κ*B and significantly decreased expression levels of I*κ*B*α* compared with control HUVECs (Figure 4(c)). These results imply that knockdown of IRSp53 exacerbates the HG-induced inflammatory state of HUVECs. In addition, neither overexpression nor knockdown of IRSp53 affected the anti-inflammatory role of insulin in the HG-induced inflammatory state (Figures 4(b) and 4(c)). The results of cellular immunofluorescence staining of NF-*κ*B further validated the conclusions regarding the anti-inflammatory role of IRSp53 and insulin (Figure 4(d)).

### 3.5. The Effect of IRSp53 on HUVEC Mobility

**Figure 5:**
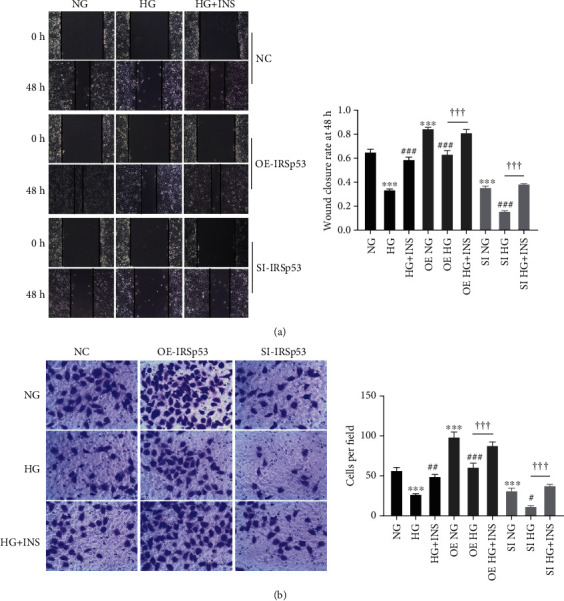
The effect of IRSp53 on HUVEC mobility. (a) Representative images of a scratch assay of HUVECs with different treatments (100x magnification) and the wound closure rate of HUVECs with different treatments. (b) Representative images of a transwell assay of HUVECs with different treatments (200x magnification) and the number of migrated cells. NC: normal control; OE-IRSp53: overexpressing IRSp53; SI-IRSp53: knockdown IRSp53; normal glucose (25 mM); HG: high glucose (60 mM); HG+INS: high glucose (60 mM) + insulin (1.5 *μ*g/mL). The values are the mean ± SD of three independent experiments. ^∗∗∗^*P* < 0.001 vs. the NG group; ^#^*P* < 0.05 vs. the HG group; ^##^*P* < 0.01 vs. the HG group; ^###^*P* < 0.001 vs. the HG group; ^†††^*P* < 0.001.

In the scratch assays, the migration of HUVECs treated with HG was significantly decreased compared with that of NG-treated HUVECs. Cotreatment with insulin reduced the HG-induced mobility impairment of HUVECs (Figure 5(a)). The results of a migration assay also validated these findings (Figure 5(b)). The effect of IRSp53 on HUVEC migration was also explored. The scratch assay and migration assay showed that the migration of IRSp53-overexpressing HUVECs was significantly increased compared with that of the control HUVECs. In addition, overexpression of IRSp53 partially protected HUVECs from HG-induced impaired migration (Figures 5(a) and 5(b)). In contrast, IRSp53-knockdown HUVECs exhibited a deteriorated capability of migration compared with control HUVECs, and IRSp53 knockdown exacerbated HG-induced migration impairment compared with the case of control HUVECs treated with HG (Figures 5(a) and 5(b)). In addition, neither overexpression nor knockdown of IRSp53 influenced the role of insulin in increasing the mobility of HG-treated HUVECs (Figures 5(a) and 5(b)).

## 4. Discussion

This study is aimed at exploring the role of IRSp53 in hyperglycemia-induced endothelial dysfunction. HUVECs were treated with HG to simulate hyperglycemia in patients with DM. The HUVECs exhibited an inflammatory state and impaired mobility after HG treatment, and cotreatment with insulin ameliorated HG-induced changes in HUVECs. In addition to these changes, HG treatment significantly downregulated the expression level of IRSp53 while upregulating that of gal-3. The overexpression and knockdown of IRSp53 in HUVECs led to reversed changes in the expression level of gal-3. In terms of endothelial function, IRSp53 overexpression downregulated the expression level of NF-*κ*B but upregulated that of I*κ*B*α* compared with the case of control HUVECs and prevented the HG-induced inflammatory state. HUVEC mobility was increased due to IRSp53 overexpression, which also prevented HG-induced mobility impairment of HUVECs. In contrast, knockdown of IRSp53 resulted in an inflammatory state of HUVECs and exacerbated HG-induced activation of NF-*κ*B. HUVEC mobility was also degraded by IRSp53 knockdown, which also intensified the HG-induced mobility impairment of HUVECs. However, neither overexpression nor knockdown of IRSp53 affected the protective role of insulin in endothelial dysfunction.

The vascular endothelium of patients with DM was exposed to hyperglycemic conditions, which promote endothelial dysfunction or even macrovascular and microvascular complications of DM [21]. The accumulation of reactive oxygen species (ROS) in endothelial cells (ECs) is an important mechanism underlying hyperglycemia-induced endothelial dysfunction [22]. ROS are capable of downregulating nitric oxide and activating NF-*κ*B, leading to dysregulated vasodilation and an inflammatory state of ECs [22, 23]. As a substrate for insulin receptor tyrosine kinase [24], IRSp53 is involved in actin cytoskeleton remodeling, filopodia formation, and membrane dynamics [25]. However, the role of IRSp53 in hyperglycemia-induced endothelial dysfunction remains unknown. In this study, HUVECs exhibited an inflammatory state and impaired mobility after HG treatment, which implicated the degradation effect of HG on ECs. In addition, HG treatment induced downregulation of IRSp53 and upregulation of gal-3 in HUVECs. STZ-induced DM rats also showed the same changes in the expression levels of IRSp53 and gal-3. These results indicated that IRSp53 might play a crucial role in hyperglycemia-induced endothelial dysfunction.

IRSp53-overexpressing HUVECs and IRSp53-knockdown HUVECs were used to further explore the role of IRSp53 in hyperglycemia-induced endothelial dysfunction. Overexpression of IRSp53 led to the downregulation of NF-*κ*B and the upregulation of I*κ*B*α* in the HUVECs. NF-*κ*B is one of the most important inflammatory mediators in humans [26]. Under physiological conditions, NF-*κ*B is retained in the cytoplasm and stays inactivated [27]. Proinflammatory stimulation can activate the I*κ*B kinase (IKK) complex, which results in phosphorylation and degradation of I*κ*B*α*, which covers the nuclear localization sequence of NF-*κ*B. Subsequently, NF-*κ*B translocates into the nucleus and binds to its target genes [27]. The changes in the expression levels of NF-*κ*B and I*κ*B*α* in IRSp53-overexpressing HUVECs suggested that IRSp53 inhibits NF-*κ*B activation. When treated with HG, IRSp53 overexpression in HUVECs could also relieve HG-induced activation of NF-*κ*B. These results implied that IRSp53 might be an anti-inflammatory factor in HUVECs and protect these cells from the inflammatory state. Furthermore, IRSp53 knockdown led to an inflammatory state of HUVECs and exacerbated the HG-induced activation of NF-*κ*B. NF-*κ*B activation is known to stimulate the transcription of inflammation-associated genes including vascular cell adhesion molecule-1 (VCAM-1), monocyte chemoattractant protein-1 (MCP-1), interleukin-1 (IL-1), and tumor necrosis factor alpha (TNF-*α*) [27]. Furthermore, the upregulation of MCP-1 in EC can recruit monocytes in circulation to EC, the upregulation of VCAM-1 facilitates the adhesion of monocytes to EC, and inflammatory factors such as IL-1 and TNF-*α* can induce monocyte differentiation into macrophages [28]. These changes significantly accelerate the process of atherosclerosis, representing the complicating macrovascular effects of DM [28]. These findings further validate that IRSp53 acts as an anti-inflammatory factor and that IRSp53 downregulation can increase the susceptibility of HUVECs to the inflammatory state. The overexpression of IRSp53 also significantly decreased the expression level of gal-3 in HUVECs. Gal-3 is secreted mainly by macrophages in circulation and is associated with inflammatory diseases and DM [19]. In acute inflammatory diseases such as pneumonia, gal-3 acts as a proinflammatory factor [29], while in chronic inflammatory diseases such as chronic pancreatitis, gal-3 promotes wound healing and fibrosis [30]. However, the role of gal-3 in DM remains controversial. Li et al. reported that gal-3 was capable of inducing cellular and systemic insulin resistance [19], while Darrow and Shohet found that gal-3 deficiency exacerbated metabolic disorders [31]. In the present study, the expression level of gal-3 was upregulated in HG-induced endothelial dysfunction. We speculated that the change in the gal-3 expression level might be associated with the inflammatory state of endothelial dysfunction. However, the exact role of gal-3 in hyperglycemia-induced endothelial dysfunction needs further exploration.

The mobility of EC significantly contributes to the integrity of the vascular endothelium, which is essential for modulating inflammation and coagulation *in vivo* [28, 32]. In addition to the inflammatory state, HG also impaired HUVEC mobility. Overexpression of IRSp53 could significantly increase HUVEC mobility and reverse the HG-induced mobility impairment of HUVECs. On the other hand, knockdown of IRSp53 had a degraded effect on HUVEC mobility and exacerbated the HG-induced mobility impairment of HUVECs. These findings indicated that IRSp53 effectively provoked the migration of HUVECs. IRSp53 is an important modulator of the actin cytoskeleton that regulates cell mobility and membrane dynamics [13]. In addition to stimulating neurite outgrowth [7], IRSp53 positively regulates the migration and invasion of cancer cells by modulating actin formation [33]. Kaur et al. reported that IRSp53 is associated with EC migration [14], but the exact role remains unknown. In this study, the results of a scratch assay and migration assay showed that IRSp53 could effectively increase HUVEC mobility.

This study also demonstrated that insulin is capable of ameliorating the HG-induced inflammatory state and HUVEC mobility impairment. Furthermore, neither overexpression nor knockdown of IRSp53 affected the protective role of insulin, implying that IRSp53 might not associate with the insulin signaling pathway in HUVECs. These results were to some extent consistent with previous research that indicated that IRSp53 knockdown in YY-8103 and Huh-7 cells does not affect the insulin signaling pathway [34]. The exact mechanism underlying the IRSp53-induced inflammatory state and impaired mobility of HUVECs needs further exploration.

## 5. Conclusions

In summary, IRSp53 was associated with HG-induced endothelial dysfunction. The overexpression and knockdown of IRSp53 in HUVECs suggested that IRSp53 acted as an anti-inflammatory factor and played a protective role in HUVEC mobility. Knockdown of IRSp53 upregulated the expression level of gal-3, which might be associated with the inflammatory state of HUVECs. Therefore, IRSp53 might be a potential therapeutic target in macrovascular and microvascular complications of DM.

## Data Availability

The data used to support the findings of this study are included within the article.

## Supplementary Materials

Supplementary Materials Descriptions of the IRSp53-siRNA and IRSp53-overexpressing lentiviruses used in this study.

## References

[1] Diagnosis ECot, Classification of Diabetes M (2003). Report of the expert committee on the diagnosis and classification of diabetes mellitus. *Diabetes Care*.

[2] Wild S., Roglic G., Green A., Sicree R., King H. (2004). Global prevalence of diabetes: estimates for the year 2000 and projections for 2030. *Diabetes Care*.

[3] Tousoulis D., Kampoli A.-M., Stefanadis C. (2012). Diabetes mellitus and vascular endothelial dysfunction: current perspectives. *Current Vascular Pharmacology*.

[4] Lavin D. P., White M. F., Brazil D. P. (2016). IRS proteins and diabetic complications. *Diabetologia*.

[5] Capellini V. K., Celotto A. C., Baldo C. F. (2010). Diabetes and vascular disease: basic concepts of nitric oxide physiology, endothelial dysfunction, oxidative stress and therapeutic possibilities. *Current Vascular Pharmacology*.

[6] DeFronzo R. A., Ferrannini E. (1991). Insulin resistance. A multifaceted syndrome responsible for NIDDM, obesity, hypertension, dyslipidemia, and atherosclerotic cardiovascular disease. *Diabetes Care*.

[7] Ahmed S., Goh W. I., Bu W. (2010). I-BAR domains, IRSp53 and filopodium formation. *Seminars in Cell & Developmental Biology*.

[8] Choi J., Ko J., Racz B. (2005). Regulation of dendritic spine morphogenesis by insulin receptor substrate 53, a downstream effector of Rac1 and Cdc42 small GTPases. *The Journal of Neuroscience*.

[9] Bobsin K., Kreienkamp H.-J. (2016). Severe learning deficits of IRSp53 mutant mice are caused by altered NMDA receptor-dependent signal transduction. *Journal of Neurochemistry*.

[10] Celestino-Soper P. B. S., Shaw C. A., Sanders S. J. (2011). Use of array CGH to detect exonic copy number variants throughout the genome in autism families detects a novel deletion in TMLHE. *Human Molecular Genetics*.

[11] Fromer M., Pocklington A. J., Kavanagh D. H. (2014). De novo mutations in schizophrenia implicate synaptic networks. *Nature*.

[12] Funato Y., Terabayashi T., Suenaga N., Seiki M., Takenawa T., Miki H. (2004). IRSp53/Eps8 complex is important for positive regulation of Rac and cancer cell motility/invasiveness. *Cancer Research*.

[13] Liu P. S., Jong T. H., Maa M. C., Leu T. H. (2010). The interplay between Eps8 and IRSp53 contributes to Src-mediated transformation. *Oncogene*.

[14] Kaur S., Samant G. V., Pramanik K. (2008). Silencing of directional migration in roundabout4 knockdown endothelial cells. *BMC Cell Biology*.

[15] Stary H. C. (2000). Natural history and histological classification of atherosclerotic Lesions. *Arteriosclerosis, Thrombosis, and Vascular Biology*.

[16] Menini S., Iacobini C., Blasetti Fantauzzi C., Pesce C. M., Pugliese G. (2016). Role of galectin-3 in obesity and impaired glucose homeostasis. *Oxidative Medicine and Cellular Longevity*.

[17] Yang R. Y., Liu F. T. (2003). Galectins in cell growth and apoptosis. *Cellular and Molecular Life Sciences*.

[18] Yilmaz H., Cakmak M., Inan O., Darcin T., Akcay A. (2015). Increased levels of galectin-3 were associated with prediabetes and diabetes: new risk factor?. *Journal of Endocrinological Investigation*.

[19] Li P., Liu S., Lu M. (2016). Hematopoietic-derived galectin-3 causes cellular and systemic insulin resistance. *Cell*.

[20] Tan K. C. B., Cheung C.-L., Lee A. C. H., Lam J. K. Y., Wong Y., Shiu S. W. M. (2018). Galectin-3 is independently associated with progression of nephropathy in type 2 diabetes mellitus. *Diabetologia*.

[21] Naudi A., Jove M., Ayala V. (2012). Cellular dysfunction in diabetes as maladaptive response to mitochondrial oxidative stress. *Experimental Diabetes Research*.

[22] Creager M. A., Lüscher T. F., Cosentino F., Beckman J. A. (2003). Diabetes and vascular disease: pathophysiology, clinical consequences, and medical therapy: part I. *Circulation*.

[23] Giacco F., Brownlee M. (2010). Oxidative stress and diabetic complications. *Circulation Research*.

[24] Böhni R., Riesgo-Escovar J., Oldham S. (1999). Autonomous control of cell and organ size by CHICO, a Drosophila homolog of vertebrate IRS1-4. *Cell*.

[25] Carman P. J., Dominguez R. (2018). BAR domain proteins—a linkage between cellular membranes, signaling pathways, and the actin cytoskeleton. *Biophysical Reviews*.

[26] Libby P. (2002). Inflammation in atherosclerosis. *Nature*.

[27] Collins T., Cybulsky M. I. (2001). NF-*κ*B: pivotal mediator or innocent bystander in atherogenesis?. *The Journal of clinical investigation.*.

[28] Gimbrone M. A., Garcia-Cardena G. (2016). Endothelial cell dysfunction and the pathobiology of atherosclerosis. *Circulation Research*.

[29] Farnworth S. L., Henderson N. C., MacKinnon A. C. (2008). Galectin-3 reduces the severity of pneumococcal pneumonia by augmenting neutrophil function. *The American Journal of Pathology*.

[30] Wang L., Friess H., Zhu Z. (2000). Galectin-1 and galectin-3 in chronic pancreatitis. *Laboratory Investigation*.

[31] Darrow A. L., Shohet R. V. (2015). Galectin-3 deficiency exacerbates hyperglycemia and the endothelial response to diabetes. *Cardiovascular Diabetology*.

[32] Chen Y., Liu F., Han F., Lv L., Tang C.-E., Luo F. (2020). Omentin-1 ameliorated free fatty acid-induced impairment in proliferation, migration, and inflammatory states of HUVECs. *Cardiology Research and Practice*.

[33] Scita G., Confalonieri S., Lappalainen P., Suetsugu S. (2008). IRSp53: crossing the road of membrane and actin dynamics in the formation of membrane protrusions. *Trends in Cell Biology*.

[34] Huang L. Y., Wang Y. P., Wei B. F. (2013). Deficiency of IRTKS as an adaptor of insulin receptor leads to insulin resistance. *Circulation Research*.

