# Supplementary material for: Insulin Receptor Substrate p53 Ameliorates High-Glucose-Induced Activation of NF-κB and Impaired Mobility of HUVECs
Source: Biomed Res Int. 2021 Jan 6;2021:3210586. doi: 10.1155/2021/3210586 (PMC7806382; doi:10.1155/2021/3210586)
Supplement: Supplementary Materials — Descriptions of the IRSp53-siRNA and IRSp53-overexpressing lentiviruses used in this study. [file 3210586.f1.docx]

| 5′-CCA GUC CCU UGA UCG AUA UTT-3′ |
| --- |
| 5′-AUA UCG AUC AAG GGA CUG GTA-3′ |

Supplementary Table 1: the sequence of IRSp53-siRNA

IRSp53-siRNA was designed and produced by RiboBio Co., Ltd. (Guangdong China). HUVEC was transfected with IRSp53-siRNA according to the manufacturer’s recommended procedures.

The virus stock solution was synthesized by Shanghai Gemma Gene Co., LTD.
